# Good Things for Those Who Wait: Predictive Modeling Highlights Importance of Delay Discounting for Income Attainment

**DOI:** 10.3389/fpsyg.2018.01545

**Published:** 2018-09-03

**Authors:** William H. Hampton, Nima Asadi, Ingrid R. Olson

**Affiliations:** ^1^Department of Psychology, College of Liberal Arts, Temple University, Philadelphia, PA, United States; ^2^Decision Neuroscience, College of Liberal Arts, Temple University, Philadelphia, PA, United States; ^3^Computer Science, College of Science and Technology, Temple University, Philadelphia, PA, United States

**Keywords:** income, salary, delay discounting, predictive modeling, machine learning

## Abstract

Income is a primary determinant of social mobility, career progression, and personal happiness. It has been shown to vary with demographic variables like age and education, with more oblique variables such as height, and with behaviors such as delay discounting, i.e., the propensity to devalue future rewards. However, the relative contribution of each these salary-linked variables to income is not known. Further, much of past research has often been underpowered, drawn from populations of convenience, and produced findings that have not always been replicated. Here we tested a large (*n* = 2,564), heterogeneous sample, and employed a novel analytic approach: using three machine learning algorithms to model the relationship between income and age, gender, height, race, zip code, education, occupation, and discounting. We found that delay discounting is more predictive of income than age, ethnicity, or height. We then used a holdout data set to test the robustness of our findings. We discuss the benefits of our methodological approach, as well as possible explanations and implications for the prominent relationship between delay discounting and income.

## Introduction

Money is critically important in shaping human happiness and well-being. Income is a key predictor of social mobility (Griffin and Alexander, 1978) and a proxy for career progression and occupational success (Mitchell et al., 1975). In epidemiology, it has been shown that decreasing levels of income correlate with increases in morbidity and mortality, as well as a range of health problems such as heart disease, diabetes, and obesity (Epstein, 2010). Psychiatric disorders are influenced by income, such that lower income correlates with higher rates of depression and substance abuse (Zimmerman and Katon, 2005).

Rate of return from investments in higher education is also frequently measured in terms of income (Hunt, 1963). Indeed, many individuals are influenced by salary outcome when pursuing additional education and choosing degree programs (Dreher et al., 1985). Similarly income has also been associated with post-college job choice (Schoenfelder and Hantula, 2003). Perhaps most importantly, income is an important predictor of happiness and life satisfaction (Blanchflower and Oswald, 2004). Emotional well-being also positively correlates with income, although this effect diminishes among the highest earners (Kahneman and Deaton, 2010). In addition to these correlational findings, there is some evidence that higher income causes greater levels of happiness (Powdthavee, 2010).

These observations give rise to a simple yet essential question: why do some individuals make more money than others? Prior research has shown that salary varies with educational attainment, cognitive ability, and broad measures of socioeconomic status (de Wolff and Van Slijpe, 1973; Ceci and Williams, 1997; Roberts et al., 2007), in addition to varying with several demographic and physical variables such as where one lives (Internal Revenue Service, 2014), gender (Nadler et al., 2016), race (Castilla, 2008), and even height (Judge and Cable, 2004).

Other researchers have attempted to use behavioral variables to predict salary, the most famous example being derived from the well-known Marshmallow Test. Walter Mischel and colleagues found that children who exhibited greater self-control on a simple, one-shot delay discounting task that involved the choice between getting one treat now or more treats if they were willing to wait a few minutes, were more likely to have higher salaries later in life (Casey et al., 2011).

Together, this work has provided a basis for our understanding of which traits and behaviors relate to income attainment. However, previous research has been limited in several ways. First, much behavioral research has been underpowered, raising a myriad of well-documented concerns regarding scientific rigor (Francis, 2012). Second, psychologists have often drawn too heavily on undergraduate sample populations from universities that tend to be Caucasian, well-educated, and relatively wealthy. The use of participants from this so-called “W.E.I.R.D.” population (Henrich et al., 2010) has therefore limited the generalizability of many previous findings. With these two concerns in mind, we captured delay discounting behavior as well as an array of demographic information from a large (*n* = 2,564) adult sample that was heterogeneous in terms of age, education, income, ethnicity, and race. For practical reasons, we collected this large, heterogeneous sample via an online protocol.

A related set of issues arises from methodological approach, which is partially responsible for the proliferation of “sexy” findings that do not replicate (Fiedler, 2017). To address this, we confirmed our results using 10-fold cross-validation. By iteratively testing our findings from one subset of the data on the remaining data, we test the robustness of our results and mitigate pitfalls such as overfitting that can hamper more traditional statistical approaches such as linear regression (Hurvich and Tsai, 1989; Seber and Lee, 2012).

Finally, although previous studies have identified an array of factors that relate to income, no study has modeled this large number of contributing variables simultaneously, leaving the relative importance of each predictive variable unclear. For instance, although both gender and discounting are known to relate to salary, it is not known which is *more* predictive of one’s salary. Further, this variable set exhibits multicollinearity, i.e., the variables that correlate with salary also relate to each other. For example, education relates to zip code (Drewnowski et al., 2007) and gender relates to height (Costa-Font and Gil, 2008). There are many possible reasons for this overlap, mostly notably that many of these variables are proxies for a more encompassing measure: socioeconomic status. This multicollinearity among variables is yet another reasons why standard correlational and regression analytic approaches are suboptimal. To address this issue, we used three types of machine learning algorithms [support vector machine (SVM), neural network, and random forest] to rank order the importance of our predictors for income. This machine learning approach is preferable to more traditional correlational or regression analyses for several reasons. First, several categorical variables such as occupation cannot be accurately modeled with traditional methods. Specifically, to include occupation in a linear regression would require dummy coding that would introduce nearly 300 binary variables into the model. The zip code variable would introduce similar issues. Second, certain machine learning algorithms, such as random forest are less sensitive to outliers (Hodge and Austin, 2004). Finally, traditional measures can only capture linear relationships between income and predictor variables; our machine learning approach allows us to model both linear and non-linear relationships. To minimize the bias associated with any one predictive model, we averaged their output rankings.

Our results show a consistent ranking of the relative importance of key variables for predicting individual differences in income, with discounting behavior occupying a position of prominence. We discuss the benefits of our methodological approach, as well as the possible explanations and implications for this robust relationship between impulsivity and income.

## Materials and Methods

### Participants

Three thousand participants (1,314 males, *M*_age_ = 41 years, range 25–65) completed the experimental protocol, which included a behavioral task and demographic questionnaire. This sample size was chosen to decrease bias and enhance the accuracy and generalizability of our analysis. All portions of the study were mandatory and thus completed by every participant. Participants were recruited through Amazon Mechanical Turk in two separate phases. In the first phase, 2,000 participants who were United States citizens and over the age of 25. The age distribution of the participants in the first phase was skewed toward younger adults. Given our goal was to have a sample representative of the United States adult population, we collected a second dataset of 1,000 participants, which included only individuals aged 35 or older. Education levels ranged from pre-high school to doctorate degree. Further information about our sample can be found the **“Methodological Detail Appendix”**.

After removing outliers (see *Outlier Removal*), 2,564 participants were used for further analysis. Informed consent was obtained according to the guidelines of the Institutional Review Board of Temple University, which reviewed and approved the experimental protocol, and affirmed ethical treatment of human participants.

### Measures

#### Delay Discounting Task

We created a Python web-based application that interfaced with Amazon’s Mechanical Turk. Participants engaged in a delay discounting task adapted from O’Brien et al. (2011). In the task, participants were asked to make choices between a smaller sum of money offered now versus a larger sum of money (always $1,000) offered at five different delays. The initial immediate reward offer was $500 for all delay periods. The delay periods were 1 day, 1 week, 1 month, 6 months, and 1 year. If the immediate reward was chosen on a given trial, the next question presented an immediate reward halfway between the prior immediate reward value and zero (i.e., a lower amount). If the delayed reward was chosen, the next question was an immediate reward midway between the prior immediate reward and $1,000. This narrowing pattern continued, with all subsequent questions presenting immediate values midway between the reward rejected and the previously rejected higher or lower reward, until participants choices converged on an indifference point to the nearest dollar, i.e., the dollar amount subjectively equivalent to the discounted delayed reward if the value were offered immediately (Ohmura et al., 2006).

Lower indifference points indicate increased devalćation of delayed rewards in favor of immediate rewards. In other words, a lower indifference point indicates higher reward impulsivity. A large corpus of research has shown that people generally discount future rewards hyperbolically, according to how long they must wait (Kirby and Maraković, 1995). Critically, there is substantial individual variability in the extent to which people discount (Myerson and Green, 1995). In this case, all rewards were hypothetical, but participants were asked to answer as if they were real. Use of hypothetical choices in a delay discounting task has been shown to yield no systematic difference in discount rate compared to real choices, suggesting that hypothetical rewards are valid proxy for real rewards (Johnson and Bickel, 2002).

#### Demographic Measurements

After completing the delay discounting task participants completed a demographics questionnaire. Participants reported their annual income, which entailed entering their “actual annual income” into a text entry field. Participants also self-reported their level of education, age, sex, height, zip code, occupation, race, and ethnicity (**Supplementary Table S2**). For occupation, participants selected from a drop-down list of over 400 job titles (Bureau of Labor Statistics, 2015). If a participant’s exact occupation was not listed, they were instructed to select the closest available occupation. The options available to participants for level of education, sex, race, and ethnicity are detailed in the **“Methodological Detail Appendix”**.

### Analysis

**FIGURE 1:**
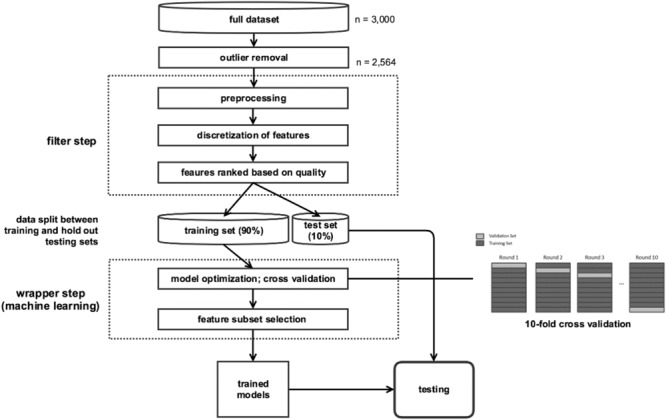
Flowchart of analytical pipeline.

We performed a multi-step analytic procedure: (1) outlier removal; (2) discretization of features (i.e., variables); (3) feature selection; (4) data split into training and testing subsets; (5) training predictive models through cross validation; and (6) measurement of predictive accuracy on unseen test data (**Figure 1**). Steps 3 through 6 were repeated iteratively, such that each tenth of the data was used once as the holdout testing set.

#### Outlier Removal

We detected outliers through extreme value detection and distribution-based inspections. We performed this outlier detection on all numeric features such as age, and height. Distributions of several features are provided in the **“Methodological Detail Appendix”**. Participants who completed the delay discounting task in less than two standard deviations (under 1.6 min) from the mean completion time were excluded. Students were also excluded from our analyses, as many active students have high levels of education yet are not employed. Outlier removal was particularly important for our correlational analyses, as well as our SVM and neural network models, all of which are sensitive to outliers (Hodge and Austin, 2004).

#### Discretization of Features

The feature vector of our dataset includes six nominal and seven numeric features. Some of the criteria that we used in our feature selection method are more compatible with categorical features. Further, reported incomes were not evenly distributed. To address this, the 313 distinct annual incomes were placed into 10 separate bins according to annual salary distribution in our sample (See **“Methodological Detail Appendix”**), such that each bin contained approximately the same number of participants. The task of predicting annual salaries based on the collected feature vectors was then converted into a multi-class classification problem, in which the goal is to predict the salary bracket to which the participants belong. This conversion also yielded a more compact representation, and thus, less complexity.

A similar discretization process was performed on the zip code data. The zip codes in our sample, which initially included over 1,700 unique values, were put into 10 separate cohorts based upon the average income in a given zip code, according to US Census Bureau (2015). As with salary data, we converted zip codes into deciles to enhance classification and feature selection precision (**Supplementary Figure S2**).

#### Feature Selection

To estimate the underlying function between the predictor features (i.e., predictor variables) and the output class (i.e., outcome variable) in a dataset, it is important to ignore the features with non-significant effect on the output (Kohavi and John, 1997). Feature subset selection (FSS) is the process of selecting the most important attribute in the feature vector for predicting the class to which each instance in the dataset belongs, in this case to find the attributes most predictive of the annual income. There are two main methods of FSS: filters and wrappers.

Filters assess feature relevance using various scoring schemes independently from a learning algorithm or classifier (Gheyas and Smith, 2010). The techniques incorporated in the filter approach are easily scalable to high-dimensional datasets, as they are computationally economical. On the other hand, wrappers, which include machine learning approaches such as neural networks, evaluate features using a specific classifier and search algorithm, where the search algorithm is wrapped around the classifier to examine the feature space (Kohavi and John, 1997). Wrapper methods consider feature dependencies and provide interaction between FSS and the choice of a learning algorithm.

Not surprisingly, both wrappers and filters each have shortcomings. For instance, although filter methods are computationally efficient due to their evaluation criteria, they do not consider the relation between the predictive model and the data. In contrast, wrapper models often result in higher predictive accuracy, but are less generalizable, and computationally more demanding (Sivagaminathan and Ramakrishnan, 2007). To account for these limitations, we used a hybrid approach for feature selection. The hybrid approach involves use of a filter as a pre-selection step followed by a wrapper stage. This hybrid approach has been shown to yield higher accuracy than a wrapper or filter alone (Sivagaminathan and Ramakrishnan, 2007).

The workflow of our approach is illustrated in **Figure 1**. A set of features was pre-selected by the filter, tested by a classifier, and then the classifier was evaluated for accuracy via validation. The most relevant features were then selected using an iterative process in which the accuracy of each classifier was calculated by removing each feature one-by-one. If removing a feature results in a decrease in accuracy of the classifier, this indicates that feature is a relevant predictor.

For our filter phase, we used the ReliefF algorithm (Robnik-Šikonja and Kononenko, 2003). For our wrapper phase, we use three validated classifier approaches: SVM, neural networks, and random forest. We selected these models because they have been widely used for classification and feature selection applications in computer science (Díaz-Uriarte and De Andres, 2006), can capture non-linearities in the dataset, are suitable for the size of our dataset, and have been successfully incorporated into hybrid approaches in the past (Huda et al., 2010). Machine learning steps were programed using the scikit-learn software package in Python (Pedregosa et al., 2011).

After the set of preselected features were obtained using the filter phase, a classifier examined the features. The accuracy of the classifier then was used to rank the importance of the remaining features. The results of the wrapper method variants were then averaged to minimize overall bias. Specifications of how each machine learning algorithm was set-up, including hyperparameter optimization, is detailed in the **“Methodological Detail Appendix”**. As noted above, we did not use linear regression for a several reasons. However, we do provide the more familiar Pearson correlations for continuous numeric attributes (**Table 1**).

**Table 1:** Pearson’s bivariate correlations among continuous variables.

	Discount 1	Discount 7	Discount 30	Discount 180	Discount 365	Age	Height	Income
**Discount 1**	–							
**Discount 7**	0.39^∗∗∗^	–						
**Discount 30**	0.34^∗∗∗^	0.61^∗∗∗^	–					
**Discount 180**	0.16^∗∗∗^	0.48^∗∗∗^	0.66^∗∗∗^	–				
**Discount 365**	0.12^∗∗^	0.40^∗∗∗^	0.57^∗∗∗^	0.79^∗∗∗^	–			
**Age**	0.05	0.04	0.02	0.06	0.04	–		
**Height**	0.04	0.12^∗∗^	0.10^∗∗^	0.09^∗∗^	0.08^∗^	0.03	–	
**Income**	0.05	0.09^∗^	0.16^∗∗∗^	0.19^∗∗∗^	0.23^∗∗∗^	0.00	0.19^∗∗∗^	–

Discounting numbers are time delay in days. ^∗^ = *p* < 0.05; *^∗∗^* = *p* < 0.01; *^∗∗∗^* = *p* < 0.001.

## Results

### Descriptive Statistics

**FIGURE 2:**
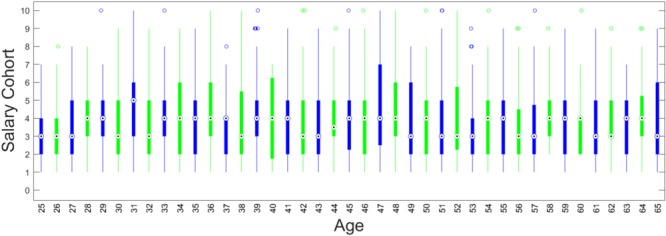
Distribution of salary by age. Each bulls-eye indicates the median value. Higher values indicate higher salaries.

Previous studies have shown that Amazon turkers tend to be more diverse than convenience college samples (Smith and Leigh, 1997), yet still younger and less ethnically diverse than the population at large (Ross et al., 2010). Our large sample of participants from MTurk was demographically heterogeneous compared to college samples. Specifically, our sample had a wide range of ages (25–65), education (pre-high school to doctorate degree) and annual income ($10,000–$235,000). Our sample was also racially and ethnically heterogeneous compared to previous samples (Sugden and Moulson, 2015). Specifically, African Americans represented 5% of the sample, Asians 6%, American Indian or Alaskan Native 2%, “other races/unknown” 4%, the remaining 83% were Caucasian, Hispanics comprised 8% of the dataset. Participants also reported their zip code, which is often used as a proxy for socioeconomic status (Krieger et al., 2002). These were recoded into income deciles (**Supplementary Table S3**) based on US Census Bureau (2015). Income varied with age, but the most prevalent income bracket was $35,201–$41,300 (See **Figure 2** and **Supplementary Figure S1**). More information about our sample is provided in the **“Methodological Detail Appendix”**.

### Correlational Findings

**FIGURE 3:**
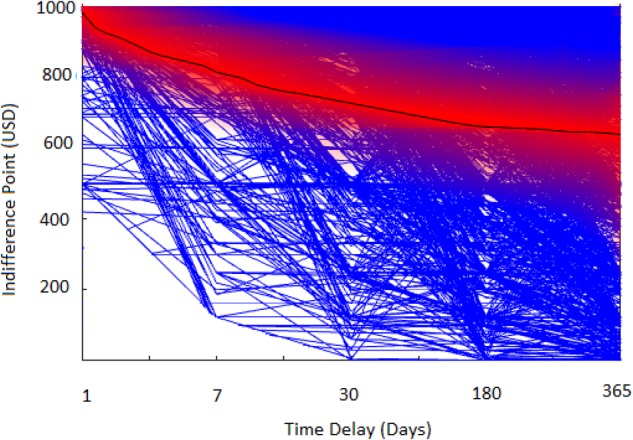
Visualization of discounting of future rewards based on points of indifference between delayed future reward ($1,000) and discounted immediate reward. Blue lines represent each study participant’s indifference function; the black line is the average discounting curve with standard error denoted by the color red.

In this study, we were interested in the relative contribution of an array of factors associated with income achievement. Prior to predictive modeling, we conducted bivariate Pearson’s correlations among our continuous variables: delay discounting, age, height, and income. We also examined the relationship between education level and annual income via a Spearman’s rank order correlation and found educational level and income were significantly correlated [*r*(2652) = 0.42, *p* < 0.001], consistent with a large corpus of prior research. Several delay discounting indifference points correlated with income, most strongly for the largest delay period, 1 year [*r*(2652) = 0.23, *p* < 0.001]. **Figure 3** shows the distribution density of the indifference points in the dataset. Consistent with prior research (e.g., Vincent, 2016), variance in discounting in our sample increased as the delay period increased. The mean value of the delay discounting attributes exhibited an inverse relationship with delay period, such that rewards further in the future were discounted more steeply. Consistent with prior research (Judge and Cable, 2004), height also significantly correlated with income [*r*(2652) = 0.19, *p* < 0.001]. It is theorized that taller height leads to greater self- and social- esteem, which leads to higher objective and subjective job performance, which in turn leads to improved career success and ultimately higher compensation. Interestingly, age did not linearly correlate with income attainment. However, further inspection of our dataset showed that age had a curvilinear relationship with income. A full matrix of these Pearson’s correlations is summarized in **Table 1**.

### Filter Results

**FIGURE 4:**
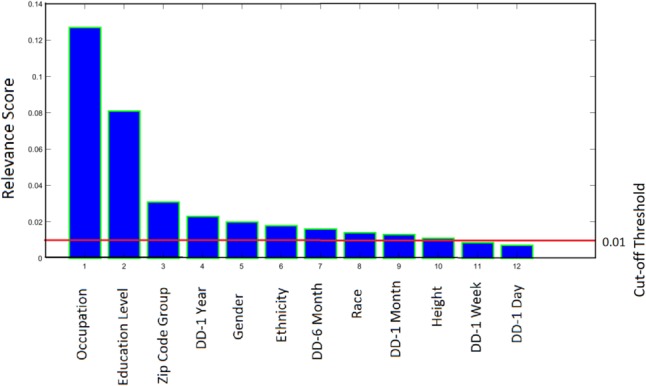
The ReliefF algorithm gives a relevance score to each feature based on its quality in predicting the class to which data points belong. DD, delay discounting.

The result of the ReliefF for the filter step indicates that occupation has the highest power in discriminating between response variables throughout the feature vector (**Figure 4**). The indifference point for the delay periods of 1 day and 1 week were the least predictive of individual income, and below the assigned threshold value. As such, these features were removed by the filter algorithm. The remaining 10 variables were included in subsequent analyses.

### Wrapper and Feature Selection Results

In this stage, we used three population machine learning models (SVM, random forest, and neural network) to simultaneously model the relationship between our predictor variables and individual income. Although rankings varied, occupation had the highest score in all three predictive models, followed by education level. Among the five delay discounting indifference attributes, the 1 year delay was most predictive of income. A full list of rankings for each model, as well as overall average ranking of each variable is summarized in **Table 2**.

**Table 2:** Attributes ranked according to how well they predicted salary.

	Support vector machine	Neural network	Random forest	Mean rank
*Occupation*	1	1	1	1
*Education*	2	2	2	2
*Zip code*	4	3	3	3.3
*Gender*	3	6	4	4.3
*1 yr. Discounting*	5	4	7	5.3
*Ethnicity*	6	8	5	6.3
*Height*	7	7	6	6.7
*6 mo. Discounting*	9	5	10	8
*Age*	8	10	9	9
*Race*	10	9	8	9

### Model Optimization, Cross Validation, and Testing

**FIGURE 5:**
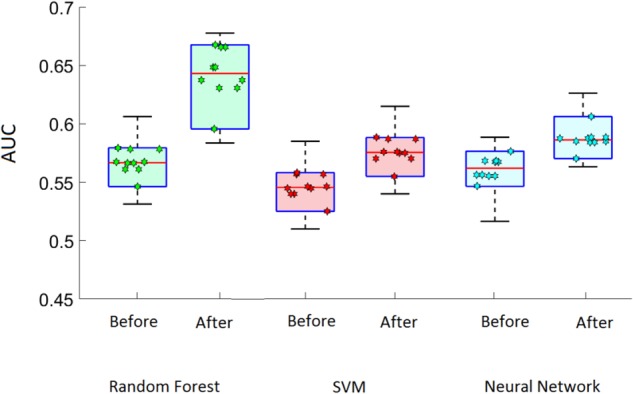
The effect of removing redundant features from the feature set over 10 trials for each model. Markers inside the box plots represent the result of each of the 10 trials. AUC, area under the curve; SVM, support vector machine. Increase in area under the curve indicates higher accuracy.

Aside from removing redundant features through the filter phase, we were able to obtain a higher classification accuracy by removing the least predictive variables (**Figure 5**). The accuracies are presented as the area under the receiver operating characteristic (ROC) curve. This measurement is particularly appropriate for demonstrating a model’s accuracy when the problem is a multi-class classification problem (Hanley and McNeil, 1982) because AUC reveals the true positive (recall) and false positive rate trade off, and is less prone to incorrect measures when dealing with imbalanced classes. Higher AUC indicates high accuracy.

This training and testing process was carried out 10 times, such that the original data (2,564 subjects) was shuffled before being separated into the subsequent training and testing sets. The result of each of these runs is quantified by the AUC, with higher AUC indicating higher accuracy. The result of each of these 10 runs is represented by the markers inside the box plots in **Figure 5**. The horizontal line in each box represents the average accuracy for each model, both before and after removing redundant features. Random Forest demonstrated the best accuracy among the three classifiers. This algorithm displayed the highest performance when its seven top performing features were employed.

## Discussion

In this study, we captured delay discounting behavior as well as an array of demographic information from a large, heterogeneous sample and found that individual differences in income are explained by a set of variables that can be ranked in a consistent manner (**Table 1**). We generated this ranking using predictive modeling, a technique commonly used in computer science that is slowly gaining popularity in psychology and neuroscience (Schwartz et al., 2013; Chang and Tsao, 2017). This technique has several advantages over other approaches. First, we were able to model continuous, categorical, and dichotomous variables simultaneously, and subsequently compare their relative importance for predicting income; this would not have been possible using more traditional methods. Second, we did not need to assume a linear relationship between income and our predictor variables. This was important for several variables, such as age, which in our large, heterogeneous sample, had a curvilinear relationship with income. This curvilinear relationship is consistent with data relating age and income from the overall United States population, as reported by the United States Government (US Bureau of Labor Statistics, 2014). This non-linear relationship is likely due to changes in pay and employment with age: the hourly wage of the typical older worker increases slightly with age for as long as he or she is employed full time, then declines upon entering partial retirement, and remains mostly flat thereafter (Becker, 1994). Finally, we used a hybrid wrapper-filter method to yield rankings from three validated machine-learning algorithms. It is worth noting that when interpreting model accuracy that it is not possible to determine which particular variable relationships are responsible for observed differences in accuracy. For instance, the slightly higher performance of the random forest model could be due to a more accurate parsing of the relationship between income and any of the predictors, such occupation. Further, the three algorithms use different mathematical criteria to make prediction: Random forest uses data entropy, SVM uses geometrical distance, and neural network uses error backpropagation. Therefore, to minimizing overall bias and maximize the information provided from these complementary analyses, we average all three resultant rankings.

The results of each model were quite consistent, with occupation and education paramount in each case. On average, the next most important factors were zip code group and gender. While zip code group was highly associated with income, it is worth noting that our data do not adjudicate directionality. Logically, a person’s income is more likely a determinant of where they live than vice versa. Nonetheless, zip codes are a useful proxy for socioeconomic status, which is also related to income (Winkleby et al., 1992). As our zip codes were binned by average income, the association between zip code and income is not surprising, but does suggest that the individuals in our sample had incomes roughly representative of the incomes from their respective zip code group. Regarding gender, we found that males earned more money than females, a result consistent with a corpus of research on the gender wage gap (Nadler et al., 2016). The fifth most important variable was delay discounting, a factor closely related, but distinct from impulsivity. Although previous research had indicated that discounting was related to income (Green et al., 1996), it was unclear to what extent, relative to other factors, this variable mattered. Interestingly, delay discounting was more predictive than age, race, ethnicity, and height (see **Supplementary Table S1**).

### Generality of Findings

Our sample was unusually diverse and inclusive for a psychology study, which have sometimes been criticized for being “W.E.I.R.D.” and unrepresentative of the general population. Our age range was large, both males and female were well-represented, educational attainment ranged from pre-high school to doctorate degrees, and more than 1,700 zip codes were represented in the final sample. However, one shortcoming of our sample is that certain minority populations were under represented relative to United States population at large. African Americans and Hispanics comprise 13 and 17% of the United States population. However, in our sample, they made up 6 and 7% of study participants, respectively.

In addition, our sample was purposely limited to Americans. It is possible that the rank order of variables that predict salary may differ in other countries. For instance, some Scandinavian countries have steeply graded income tax as well as higher levels of salary control, thereby equalizing social class differences. These countries also have some of the lowest gender pay gaps in the world. These differences would likely change the ordering of demographic variables found in **Table 2**. Nevertheless, we speculate that impulsivity, as measured by delay discounting, would continue to be a significant predictor of income attainment.

We were also limited to variables that could be collected reliably in an online protocol. There are other variables that are potentially linked to income that we did not capture in this study. For example, we did not measure intelligence, which is known to relate to income (Roberts et al., 2007). We did, however, collect level of education which has been consistently correlated with intelligence (Ceci, 1991; Neisser et al., 1996). Thus, we believe that our education variable controls for some of the variance relating to intelligence.

Finally, our approach did not seek to directly address the relationship between individual occupations and delay discounting. In our study, participants chose their occupation from a list of over 250 occupations, i.e., occupation was a nominal variable. In contrast, delay discounting was a numeric variable, rendering comparison of the two complicated. Binning occupations into broader number of categories would be highly subjective, meaning any ensuing analyses would be difficult to interpret. Future research could examine more directly the relationship between delay discounting and occupation.

### Why Does Delay Discounting Predict Income Attainment?

Our findings raise the question: why do individual differences in discounting of future rewards predict income attainment? First, it is important to note that our study was cross-sectional and therefore cannot establish casual directionality between delay discounting and income. However, we speculate that this relationship may be a consequence of the correlation between higher discounting and other undesirable life choices. For instance, higher discounting has been associated with use and abuse of addictive substances such as cigarettes (Bickel et al., 1999), alcohol (Vuchinich and Simpson, 1998), and opiates (Perry et al., 2005). Similarly, pathological gamblers have also been shown to exhibit heightened delay discounting. Inability to delay future rewards is also associated with lower intelligence (Shamosh et al., 2008), and poorer psychiatric health (Crean et al., 2000). In this way, one possibility is that delay discounting signals a cascade of negative behaviors that derail individuals from pursuing education and may ultimately preclude entry into certain lucrative occupational niches. Future longitudinal research could be designed to test this theory.

These negative behavioral outcomes, and associated steep discounting, may be partially due to reduced cognitive control (Dalley et al., 2011; Hampton et al., 2017). Difficulties delaying gratification may also be mediated by episodic future thinking, i.e., the ability to project oneself into the future to pre-experience an event (Atance and O’Neill, 2005). There is evidence that future rewards are discounted less when people engage in greater episodic future thinking (Dassen et al., 2016). Heighted or more vivid episodic future thinking is thought to induce heightened functional neural coupling of key valuation and decision-making brain areas (Peters and Büchel, 2010). Put simply, if people can vividly imagine themselves in the future with the larger rewards, they are more likely to be patient.

Whether discounting rate is a trait or a more mutable state variable is under debate. Some research has found discounting rates (Harrison and McKay, 2012) and the related ability to delay gratification to be quite stable over time (Casey et al., 2011). However, other research indicates that discounting is relatively plastic, changing as we age (Steinberg et al., 2009), and varies depending on context (Dixon et al., 2006) and state (Reynolds and Schiffbauer, 2004). If the latter perspective is accurate, then it is possible that interventions could be designed to increase cognitive control, and reduce delay discounting. Diamond and colleagues conducted such an intervention in preschool children. They found that children from low-income households who completed an executive function training curriculum exhibited improved cognitive control on a variety of tasks, and importantly, that this improved cognitive control tracked academic achievement (Diamond and Lee, 2011). It is feasible that similar interventions could be designed for adults.

In a similar vein, episodic future thinking may also be enhanced by training. As mentioned, episodic future thinking entails pre-experiencing an event in the future. This is distinct from planning, which requires multiple processing components such as problem representation, goal selection, strategy choice, and strategy execution (Atance and O’Neill, 2005). Daniel and colleagues found that merely asking participants to engage in future thinking resulted in reduced discounting, which varied according to the self-reported vividness of the imagined thoughts (Daniel et al., 2013). More research is required to determine if long-lasting changes in episodic future thinking can be obtained by training children and young adults. The possibility of early educational and training interventions could help individuals act less impulsively and be more future-oriented is an exciting and feasible prospect. Our findings suggest that such interventions could have literal payoffs in terms of higher income attainment.

## Author Contributions

WH and IO conceived the project hypotheses. WH designed the experimental protocol. NA collected all data. NA and WH conducted analyses. All authors contributed to writing of this manuscript.

## Conflict of Interest Statement

The authors declare that the research was conducted in the absence of any commercial or financial relationships that could be construed as a potential conflict of interest. The reviewer GK and handling Editor declared their shared affiliation at the time of the review.
