# Supplementary material for: Good Things for Those Who Wait: Predictive Modeling Highlights Importance of Delay Discounting for Income Attainment
Source: Front Psychol. 2018 Sep 3;9:1545. doi: 10.3389/fpsyg.2018.01545 (PMC6129952; doi:10.3389/fpsyg.2018.01545)
Supplement: Supplementary file 1 [file Data_Sheet_1.docx]

Methodological Detail Appendix

**Delay discounting task**

Below are screenshots from the delay discounting task component of the protocol.

Instructions


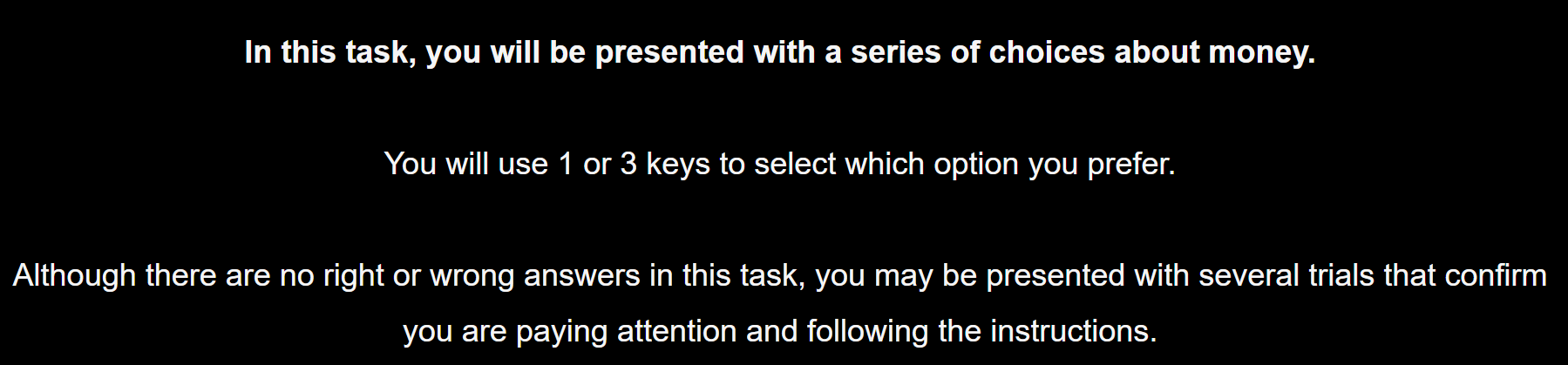


Example of forced choice between smaller sooner and larger later trial.

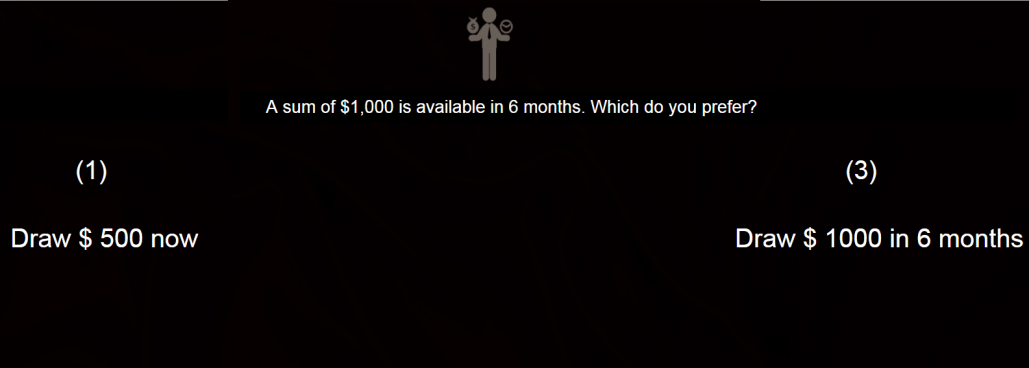


***Hyperparameter Optimization***

Hyperparameters are variables that are set before the start of training to govern the underlying learning model. The hyperparameters were determined for both the filter and wrapper algorithms through several training experiments. For the ReliefF method, the number of nearest neighbor's k (see Supplementary Information) was set to 10, and the significance threshold was set to 0.01. As mentioned previously, this entails that in each iteration 10 neighboring data points were considered for distance comparison between the attributes in different classes.

For our non-linear support vector machine algorithm, the gamma value for the radial basis function was set to 0.01. This parameter decides the bias and variance for the non-linear kernel. For the neural network used for this dataset, we had one hidden layer with maximum number of iterations set to 10000. The batch size and learning rate were set to 100 and 0.01 respectively. For the Random forest algorithm, the number of iterations and batch size were both set to 100. In calculating the classification accuracy, 10-fold training/test was performed, i.e. 90% of the data points were used for training and 10% for test phase of the data. The accuracies are the average of 10 trials. The Python Scikit-Learn library implementation of the machine learning algorithms was used for performing the experiments.

**Supplemental Tables.**


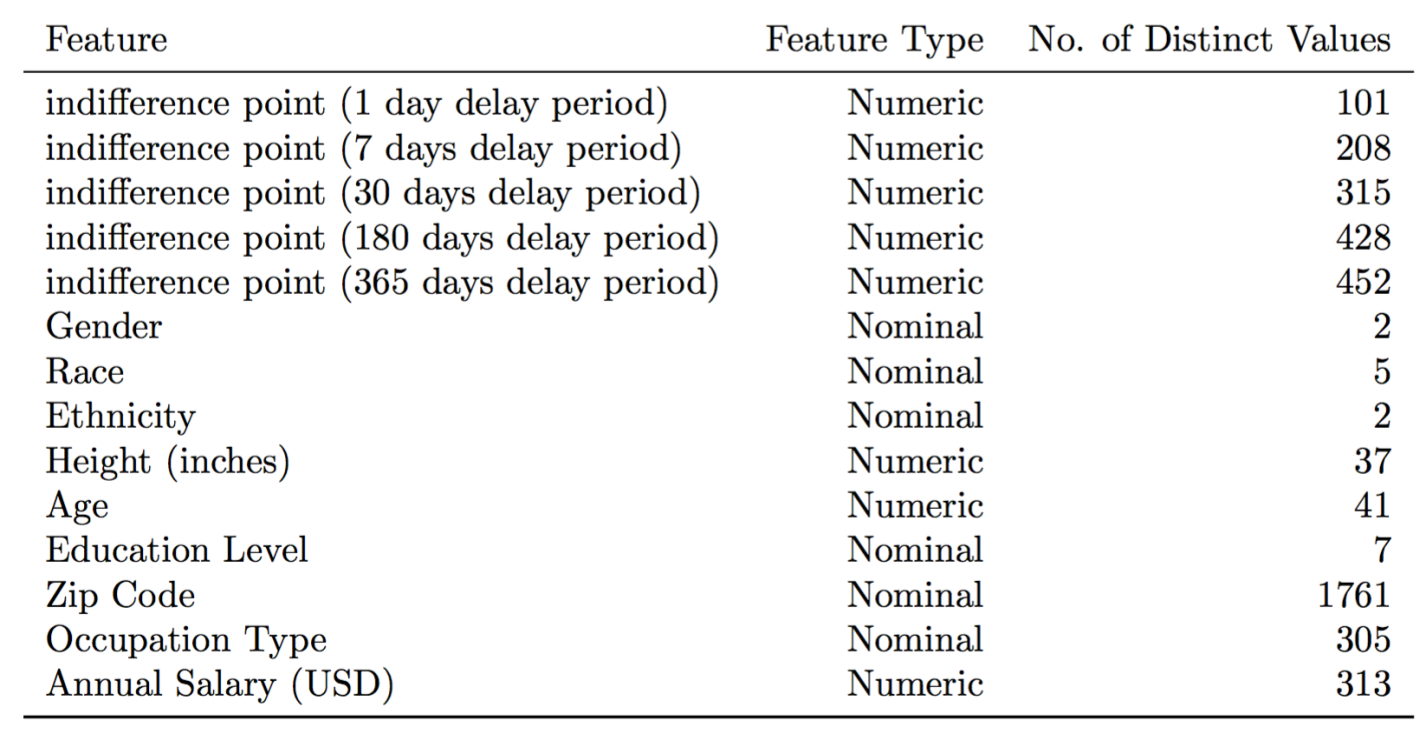


Table 1. Dataset features and characteristics


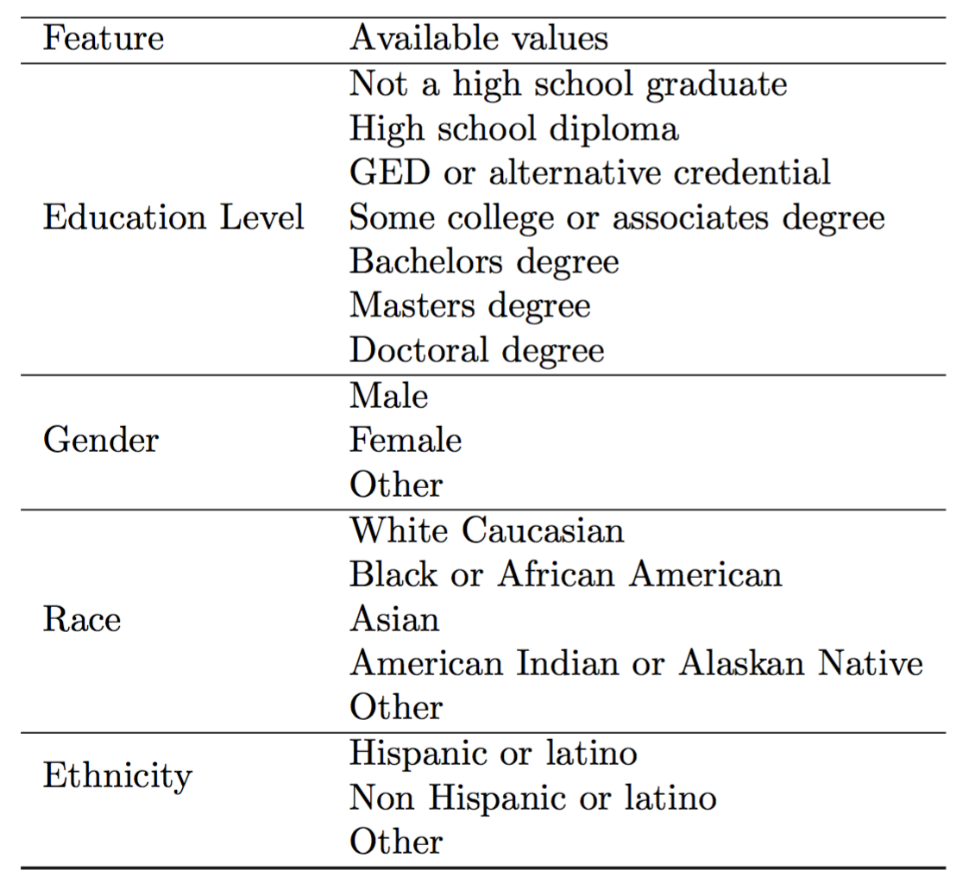


Table 2. Nominal features in the dataset and the choices available to the subjects for each feature.


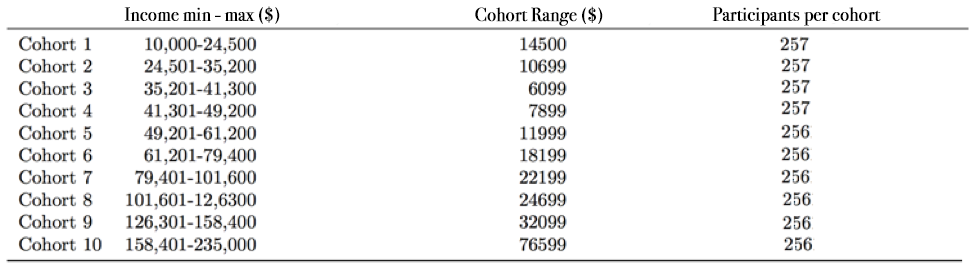


Table 3. Discretization of income into deciles.

**Supplemental Figures.**


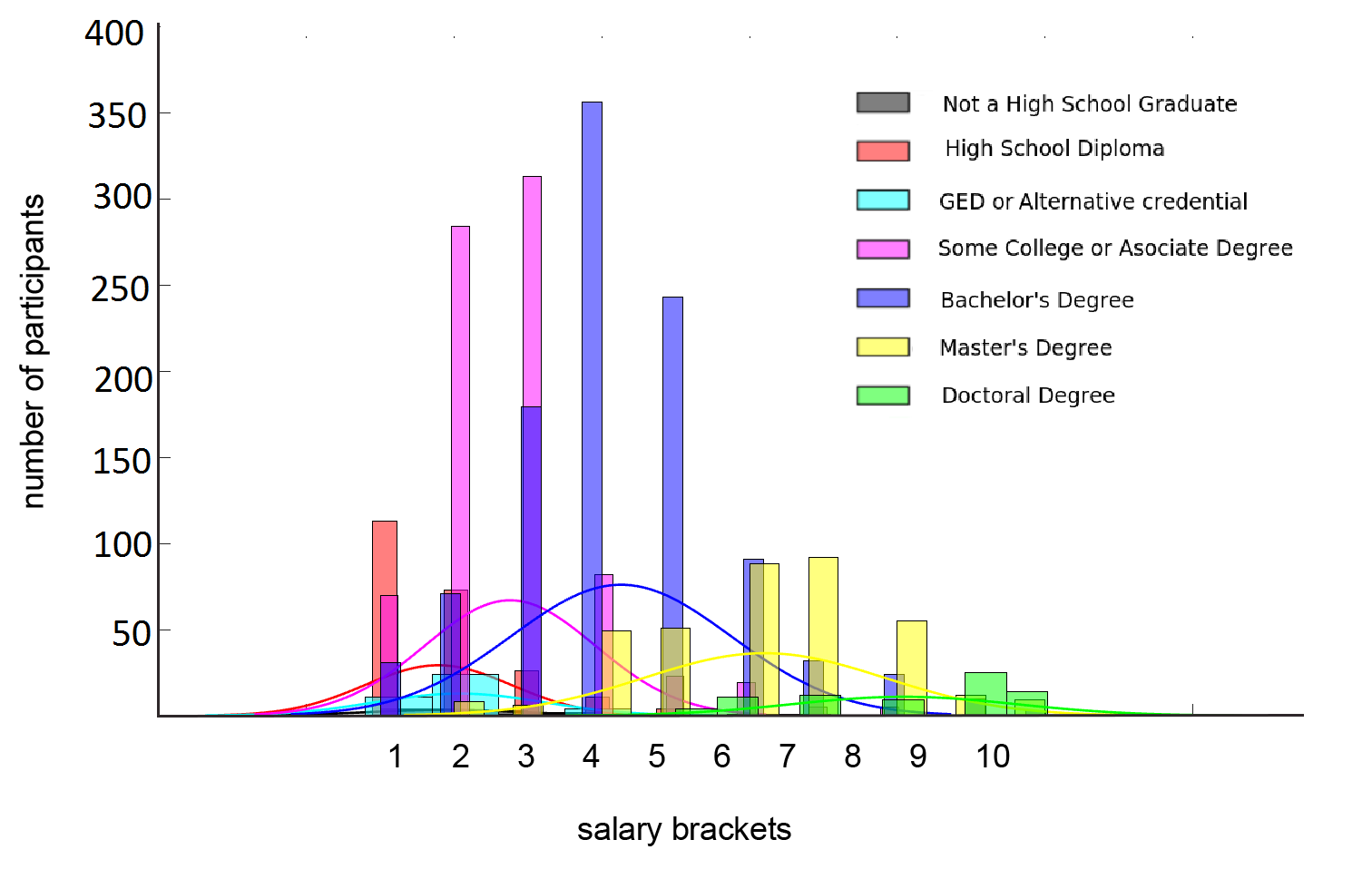


Figure 1. Distribution of participants based on income bracket and education. Salary brackets correspond to cohort number from Table 3.


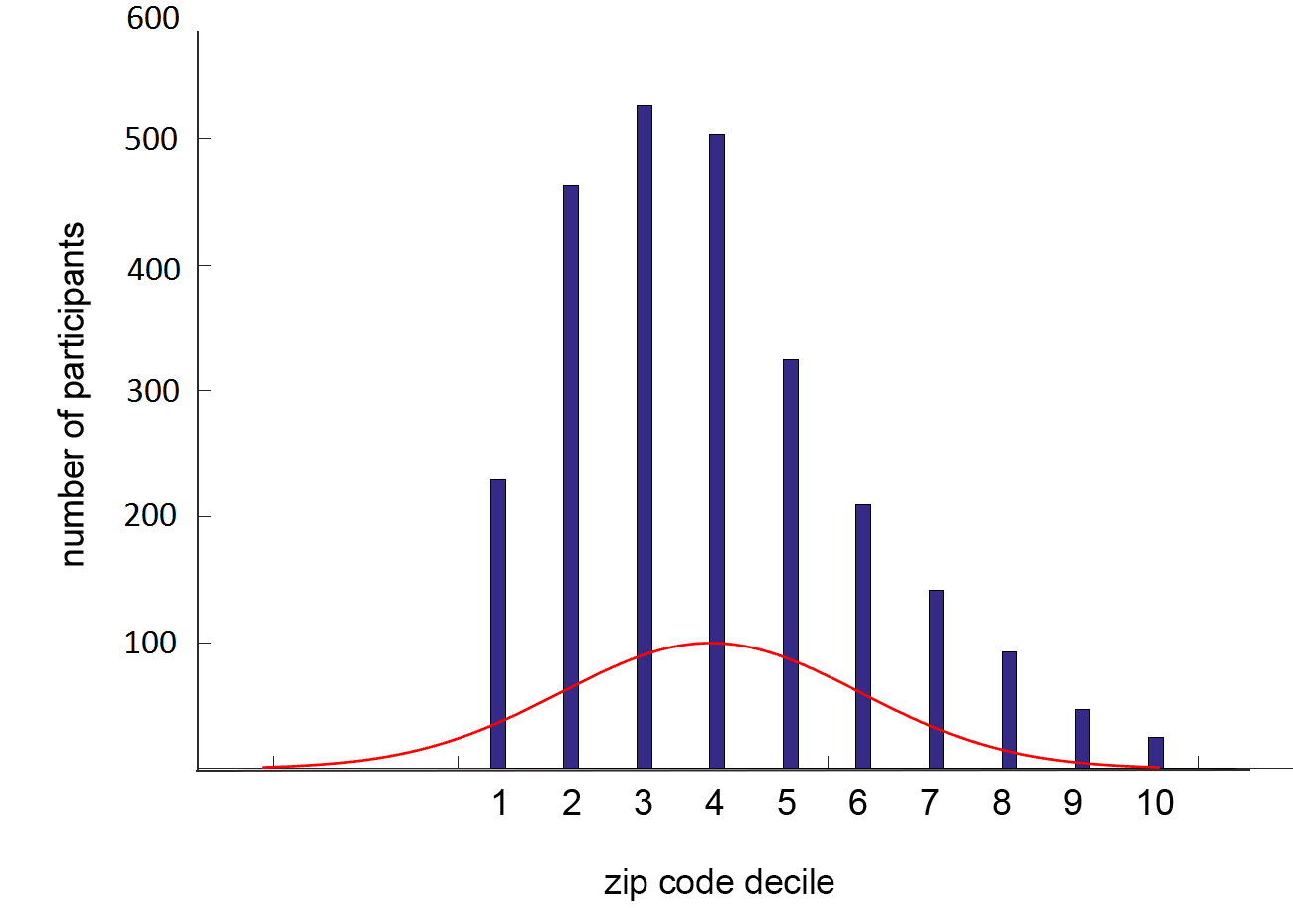


Figure 2. Discretization of zip codes into decile brackets.
